# Gonadal transcriptomics elucidate patterns of adaptive evolution within marine rockfishes (*Sebastes*)

**DOI:** 10.1186/s12864-015-1870-0

**Published:** 2015-09-02

**Authors:** Joseph Heras, Kelly McClintock, Shinichi Sunagawa, Andres Aguilar

**Affiliations:** Department of Ecology and Evolutionary Biology, University of California Irvine, 321 Steinhaus Hall, Irvine, CA 92697 USA; School of Natural Sciences and Graduate Group in Quantitative and Systems Biology, University of California Merced, 5200 N Lake Rd, Merced, CA 95344 USA; European Molecular Biology Laboratory, Meyerhofstr 1, 69117, Heidelberg, Germany; Department of Biological Sciences, California State University Los Angeles, 5151 State University Dr, Los Angeles, CA 90032 USA

**Keywords:** Bioinformatics, Orthologs, Positive selection, Reproductive genes, Untranslated region, Zona pellucida

## Abstract

**Background:**

The genetic mechanisms of speciation and adaptation in the marine environment are not well understood. The rockfish genus *Sebastes* provides a unique model system for studying adaptive evolution because of the extensive diversity found within this group, which includes morphology, ecology, and a broad range of life spans. Examples of adaptive radiations within marine ecosystems are considered an anomaly due to the absence of geographical barriers and the presence of gene flow. Using marine rockfishes, we identified signatures of natural selection from transcriptomes developed from gonadal tissue of two rockfish species (*Sebastes goodei* and *S. saxicola*). We predicted orthologous transcript pairs, and estimated their distributions of nonsynonymous (Ka) and synonymous (Ks) substitution rates.

**Results:**

We identified 144 genes out of 1079 orthologous pairs under positive selection, of which 11 are functionally annotated to reproduction based on gene ontologies (GOs). One orthologous pair of the zona pellucida gene family, which is known for its role in the selection of sperm by oocytes, out of ten was identified to be evolving under positive selection. In addition to our results in the protein coding-regions of transcripts, we found substitution rates in 3’ and 5’ UTRs to be significantly lower than Ks substitution rates implying negative selection in these regions.

**Conclusions:**

We were able to identify a series of candidate genes that are useful for the assessment of the critical genes that diverged and are responsible for the radiation within this genus. Genes associated with longevity hold potential for understanding the molecular mechanisms that have contributed to the radiation within this genus.

**Electronic supplementary material:**

The online version of this article (doi:10.1186/s12864-015-1870-0) contains supplementary material, which is available to authorized users.

## Background

Genomic information can increase our understanding of the molecular evolutionary processes that drive speciation [1]. Comparative genomic and transcriptomic studies have provided a framework for understanding how genes and genomic sequences relate to adaptation and phenotypic evolution at the organismal level [2]. Many of these comparative studies [1, 3, 4] identify coding genes that are subject to rapid divergence and positive selection, a process where mutations are advantageous and favored. Either a single mutation or an accumulation of advantageous mutations can contribute to the process of adaptive evolution. The identification of positive selection at the molecular level has been frequently estimated by the calculation of nonsynonymous (Ka) and synonymous (Ks) substitutions, in which a Ka/Ks ratio greater than one is an indication of positive selection and a value less than one is indicative of negative or purifying selection, the purging of deleterious alleles [5–7]. Genes under positive selection are generally categorized within comparative genomic studies under processes such as biosynthesis, development, metabolic processes, immune function and reproduction [1, 3, 4]. As more genomic and transcriptomic information becomes available, we can reaffirm or redefine which processes are pertinent to the processes of adaptation and speciation.

Identifying the mechanisms of speciation within marine systems has been a daunting and difficult task. Most studies, post Mayr [8], have focused on identifying geographic barriers that would prompt allopatric speciation [9]. However, within marine ecosystems there are limited geographic barriers that would prevent allopatric speciation [10]. This concept suggests a marine-speciation paradox, where incipient species that come into contact frequently would prevent allopatric speciation [11]. Rockfishes (genus *Sebastes*), an example of adaptive radiations within marine ecosystems, provide an ideal model system for understanding the mechanisms that contribute to the speciation process. The rockfish genus *Sebastes* provides a unique model system for studying adaptive evolution because of the extensive diversity found within this group, which includes variation in morphology, ecology, and a broad range of life spans [10, 12]. This rapid radiation is supported by multiple studies which demonstrate the diversification of this group from a phylogenetic context [13–15]. Ingram [10] showed evidence that rockfish speciation is associated with the divergence of habitat depth and depth-related morphology, which supports that this group of fishes are undergoing ecological speciation along an environmental gradient. Additionally, complex courtship displays and internal fertilization are found within rockfishes, making assortative mating likely [10, 16] and can help us understand how sexual selection is operating within this group.

Divergent sexual selection can facilitate the speciation process via reproductive traits that form a barrier between incipient species and result in reproductive isolation [17–19]. Other factors like spawning time, mate recognition, environmental tolerance, and gamete compatibility are thought to contribute to the marine speciation process [20]. Several molecular evolutionary studies have demonstrated that genes associated with reproduction (i.e. genes that encode for gamete recognition proteins) have rapidly diverged between closely related taxa [21–23]. Swanson and Vacquier [18] suggested that the rapid divergence within reproductive genes may stem from a single or combination of selective pressures such as sperm competition, sexual selection and sexual conflict. Levitan and Ferrell [24] demonstrated how sperm competition operates within male and female sea urchins (*Strongylocentrotus franciscanus*) in which mating pairs that had the most common bindin (sperm protein) genotypes had higher reproductive success in the presence of low polyspermy—the fertilization of an egg with multiple sperm. However, when polyspermy levels were high, males and females with unmatched bindin genotypes had the selective advantage. This depicts an “arms race” between sperm and egg proteins, in which sperm competition is a source of directional selection, and egg proteins are also under selective pressures to develop barriers against polyspermy [25]. Although a vast amount of information supports the rapid diversification of reproductive genes, very little is known about the forms of selection operating on these genes [26]. Most studies on gamete evolution within marine systems have been performed with free spawning organisms [21, 24, 26–27]. In contrast, marine rockfishes have matrotrophic viviparity, the process where the eggs are fertilized internally and the mother provides nutrition to the developing embryo and the offspring are expelled as larvae [28]. The latter is not a common life history trait in a majority of extant bony fishes. The evolutionary processes of gamete recognition proteins within this group are unknown. However, multiple paternity has been demonstrated within multiple species within *Sebastes,* including *S. goodei* [29, 30], which permits the opportunity for selective forces to operate on reproductive proteins (e.g. sperm competition and the prevention of polyspermy).

A prime candidate for understanding reproductive barriers at the molecular level is the zona pellucida (ZP) gene family, which encodes for glycoproteins that create the acellular vitelline envelope around the oocyte [31–33]. The function of ZP proteins varies in fishes and includes uptake of nutrition, functional buoyancy [34], protection of the growing oocyte, species-specific binding, and guidance of the sperm to the micropyle [35]. There are at least eight ZP genes in many fish species [36] that belong to three subfamilies: ZPB, ZPC, and ZPAX [28]. The subfamily ZPA is missing from fishes, which may be due to a gene deletion [37] and subfamilies ZPC and ZPB are known to contain gene duplicates [38]. Selection has been tested in ZPC genes in six teleost species, but the results have been inconclusive due to the lack of robustness in the statistical methods used [39]. In this study, we wanted to address more closely the hypothesis that genes in the ZP family may provide a reproductive barrier between closely related species.

Rockfishes (genus *Sebastes*) are a prime system for understanding adaptive radiations and the mechanisms of speciation within marine systems [40]. Adaptive radiations involve rapid divergence of multiple lineages, which serve as replicates of speciation within a given environment or time frame [40]. *Sebastes* spp. has been considered an ancient species flock [14], a group of closely related species with a monophyletic origin [13]. The genus arose around 8 mya, contains 22 recognized subgenera [41], and approximately 105 species found worldwide [13]. Aside from being a diverse group of fishes, there is an extensive difference in lifespans within rockfishes; the shortest-lived rockfish species is calico rockfish (*S. dalli*) at 12 years and the longest-lived rockfish is rougheye (*S. aluetianus*), which have a maximum lifespan of 205 years [28]. In addition, this genus is composed of species that are morphologically and ecologically divergent [10, 42], with the center of diversity for this group being located in the Northeast Pacific [43]. Though many studies have concentrated on describing species-level variation [13–15, 44], very few studies have investigated the genetic mechanisms that have contributed to this radiation [45, 46].

In this study, our aims were to identify and characterize genes subject to positive selection between two marine fish species in *Sebastes*. We used a comparative transcriptomic approach, in which we characterized and compared transcriptomes generated from gonadal tissues of the two species *S. goodei* and *S. saxicola*. We selected *S. goodei* (chilipepper) [47] and *S. saxicola* (stripetail) [48] based on the extensive amount of evolutionary time since their most recent common ancestor (estimated to be greater than 6 million years ago [mya] [13], which can give a broader depiction of which functional genes have diverged within this genus. In addition, gonadal tissues were selected for this study to locate highly divergent reproductive genes, which can serve as candidates for investigating positive selection across the entire genus. Our reasoning for the two different sequencing methods (Sanger and 454-pyrosequencing for *S. goodei* and *S. saxicola,* respectively) was that each library was prepared with the latest sequencing technology that was available at the time. We annotated the function of expressed genes using gene ontology (GO), and identified signatures of positive selection from estimates of Ka/Ks ratios for ortholog pairs that we annotated between these two species. Genes that were found to be evolving under positive selection were further analyzed in the context of their orthologs in model fishes and ESTs from our earlier study [46]. In addition to identifying selection through analysis of coding regions, we additionally estimated genetic divergence between the two species in untranslated regions (UTRs). Overall, this study was developed to understand how differences at the transcriptomic level contribute to adaptive evolution within this speciose group.

## Results

### Sequence statistics and annotation

The *S. goodei* ESTs contained 2370 and 13,824 raw sequences respectively and a mean EST length of 655.9 and 614 bp respectively (Additional file 1). We assembled 6139 unigenes, which were composed of 664 singletons and 630 contigs from ovary tissue, and 2849 singletons, and 1996 contigs from testes tissue. When processed through a second run of cap3 [49], the 6139 sequences were reduced to 5336 contigs and used for our comparative analyses with *S. saxicola*.

The *S. saxicola* ESTs contained a primary assembly of 311,289 reads and 295,114 clean reads. The primary assembly contained 85,431 singletons and 51,310 contigs with 71 % redundancy (Additional file 2). From these 136,741 sequences, a second assembly was processed and contained 41,174 singletons, 14,090 primary contigs, and 23,475 secondary contigs. Only sequences that were assembled into contigs and greater than 300 bp were used for our comparative analyses resulted in 3112 primary contigs and 15,393 secondary contigs were used with a total of 18,505 contigs.

There were 2480 and 8763 sequences from *S. goodei* and *S. saxicola* datasets respectively that were annotated. Within the *S. goodei* and *S. saxicola* datasets, there were Gene Ontologies (GO) terms within the biological process domain, that belonged to the cellular process, metabolic process, biological process, multicellular organismal process, developmental process, cellular component organization, response to stimulus, localization, signaling, cellular component biogenesis, reproduction, death, growth, cell proliferation, immune system process, and multi-organism process. Most GO terms represented for molecular function pertained to binding, catalytic activity, transcription regulator activity, molecular transducer activity, transporter activity, enzyme regulator activity, structural molecule activity, and electron carrier activity. The majority of GO terms represented for cellular component pertained to the cell, organelle, macromolecular complex, membrane-enclosed lumen, extracellular region, and synapse.

Our annotations of the two (*S. goodei* and *S. saxicola*) transcriptomes were relatively similar across the major three divisions (Biological Process, Molecular Function, and Cellular Component) when levels 2 and 3 GO terms were compared. In most GO terms, *S. goodei* were slightly elevated, with 2480 annotated contigs and for *S. saxicola*-8763 contigs. Although there were differences between the two sequencing methods, there were similarities in GO categories between the two transcriptomes. In addition, the two datasets showed 16 % (*S. saxicola*) and 17 % (*S. goodei*) of GO terms annotated to reproduction and 35 % and 39 % for developmental processes (respectively), which may provide an overview of reproductive processes within ovary tissues. In addition, the dual use of testes and ovary tissues from *S. goodei* contained similar GO terms between *S. saxicola* in which these two tissue types may contain similar GO functional traits.

### Genes under positive selection

**Fig. 1:**
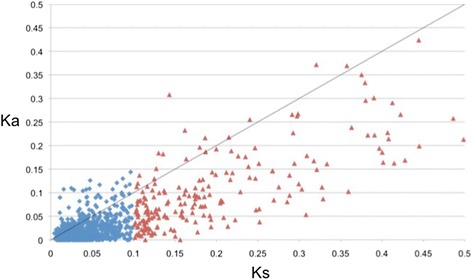
Plot of (Ka) nonsynonymous vs. (Ks) synonymous substitutions. Blue diamonds indicate values with a Ks < 0.1, whereas red triangles indicate Ks values greater than 0.1 but less than 0.5. The black line suggests neutrality, values above the line are subject to positive selection and values below are subject to purifying selection

Two hundred and nine ortholog pairs contained a Ka/Ks less than 0.1, 726 pairs were between 0.1–1.0 (Ka/Ks) and 144 pairs that were found greater than one (positive selection; Fig. 1), which amounts to 1079 orthologs in total. Seventy-one of these pairs were annotated with a majority of the sequences that were associated with macromolecule metabolic processes and regulation of biological processes based on the sequence distribution of Gene Ontologies (Table 1). Only a small fraction of the distribution of GOs were associated with reproductive process (11 orthologous pairs) and sexual reproduction (8 orthologous pairs). The average Ka/Ks value was 0.53 (s.d. = 0.62), and the average ortholog alignment length of 361.37 (s.d. = 132.45). There was no enrichment found between these two categories with a False Discovery Rate of (0.05).

**Table 1:** *S. goodei* and *S. saxicola* ortholog pairs that were identified as positive selection

*Annotation*	*Ka*	*Ks*	*Ka/Ks*	*Length*	*S. goodei Hit ACC*	*S. goodei E-value*	*S. saxicola Hit ACC*	*S. saxicola E-value*
12 kda fk506-binding protein	0.058	0.011	5.262	327	P48375	4.55E-36	P48375	2.16E-38
40s ribosomal protein x isoform	0.014	0.004	3.209	594	Q642H9	7.20E-144	N/A	N/A
60s ribosomal protein l17	0.232	0.162	1.434	153	P18621	9.36E-62	N/A	N/A
60s ribosomal protein l9	0.019	0.011	1.735	300	Q90YW0	2.53E-92	Q90YW0	6.04E-44
atp synthase mitochondrial f1 complex assembly factor 1 flags: precusor	0.032	0.02	1.583	210	Q1L987	2.27E-33	Q1L987	3.14E-81
bone morphogenetic protein 7 flags: precursor	0.011	0.01	1.124	276	P23359	1.60E-30	P23359	4.32E-45
chitobiosyldiphosphodolichol beta-mannosyltransferase	0.021	0.019	1.104	249	Q9BT22	1.46E-52	Q9BT22	5.16E-29
choline transporter-like protein 4 solute carrier family 44 member	0.029	0.021	1.368	372	Q7T2B0	3.20E-60	Q7T2B0	8.71E-80
cytochrome c oxidase subunit mitochondrial flags: precursor	0.01	0.009	1.07	423	P00426	3.68E-55	B0VYX4	5.39E-56
cytochrome p450 26a1	0.117	0.059	1.979	168	P79739	8.97E-18	P79739	9.14E-29
disintegrin and metalloproteinase domain-containing protein 9 flags: precursor	0.095	0.057	1.666	378	Q61072	2.15E-18	Q61072	1.42E-08
dna ligase 3	0.04	0.027	1.48	345	P49916	1.25E-39	N/A	N/A
dna mismatch repair protein mlh1	0.018	0.017	1.052	291	P40692	1.03E-79	P40692	3.94E-35
dna primase large subunit	0.126	0.048	2.651	354	O89044	1.47E-57	O89044	8.29E-25
double-strand-break repair protein rad21 homolog	0.13	0.087	1.493	249	O93310	4.30E-13	N/A	N/A
eukaryotic translation initiation factor 2 subunit 3	0.098	0.095	1.031	429	Q2KHU8	1.72E-110	Q2KHU8	1.35E-59
f-box only protein 11	0.081	0.067	1.204	288	Q86XK2	1.97E-49	Q86XK2	2.69E-23
f-box only protein 43 endogenous meiotic inhibitor 2	0.031	0.022	1.426	753	Q4G163	1.06E-23	Q8AXF4	2.07E-08
glioma tumor suppressor candidate region gene 2 protein	0.017	0.008	2.081	351	Q9NZM5	8.21E-33	Q9NZM5	6.12E-28
growth factor receptor-bound protein 10	0.113	0.104	1.085	216	Q13322	2.80E-47	N/A	N/A
gtpase mitochondrial	0.13	0.125	1.037	297	B5X2B8	2.46E-27	B5X2B8	1.21E-07
guanine nucleotide-binding protein g subunit alpha-2	0.09	0.06	1.491	333	P04897	3.39E-82	P04897	2.16E-43
h-2 class i histocompatibility q10 alpha chain flags: precursor	0.104	0.039	2.681	522	P01898	8.97E-44	P15979	2.66E-32
histidine triad nucleotide-binding protein 3	0.094	0.088	1.069	414	Q28BZ2	1.82E-39	Q28BZ2	7.95E-34
homolog subfamily a member 4 flags: precursor	0.037	0.029	1.289	231	Q8WW22	2.56E-49	Q8WW22	2.16E-25
importin subunit alpha-1	0.114	0.091	1.254	789	P52170	1.64E-93	P52170	2.67E-72
inositol-3-phosphate synthase 1-a	0.053	0.046	1.152	465	Q7ZXY0	3.82E-41	Q7ZXY0	5.38E-37
kelch domain-containing protein 1	0.022	0.011	2.034	318	Q8N7A1	3.47E-34	Q8N7A1	1.02E-18
lag1 longevity assurance homolog 2	0.022	0.014	1.623	258	Q96G23	2.83E-59	Q3ZBF8	6.32E-42
lamina-associated polypeptide isoform beta	0.027	0.015	1.803	432	Q62733	4.90E-10	Q62733	9.94E-09
lipid phosphate phosphohydrolase 3	0.09	0.043	2.096	201	Q3SZE3	3.77E-25	Q3SZE3	1.14E-50
map3k12-binding inhibitory protein 1	0.009	0.008	1.081	450	Q99LQ1	1.89E-38	N/A	N/A
mif4g domain-containing protein a	0.036	0.034	1.034	174	B0UXU6	2.02E-28	B0UXU6	3.33E-18
n-acetylneuraminate lyase	0.045	0.038	1.184	477	Q5RDY1	3.28E-54	Q6NYR8	8.41E-72
nad-dependent deacetylase sirtuin-5 flags: precursor	0.029	0.027	1.111	354	Q8K2C6	4.35E-65	Q3ZBQ0	2.41E-32
nuclear pore complex protein nup54	0.075	0.044	1.714	492	P70582	3.74E-33	N/A	N/A
p43 5s rna-binding protein	0.056	0.038	1.485	249	P25066	9.42E-14	P25066	4.10E-08
pentatricopeptide repeat-containing protein 2	0.012	0.011	1.073	585	Q566X6	1.63E-44	Q566X6	7.01E-97
peptidyl-prolyl cis-trans isomerase-like 2	0.018	0.008	2.303	471	Q13356	4.11E-74	Q13356	2.29E-71
poly-specific ribonuclease parn	0.019	0.016	1.2	375	O95453	4.25E-71	O95453	1.25E-61
pq-loop repeat-containing protein 2	0.029	0.018	1.605	402	Q8C4N4	2.03E-42	Q8C4N4	4.36E-41
proteasome subunit alpha type-2	0.045	0.013	3.55	423	O73672	8.15E-122	O73672	1.73E-64
protection of telomeres protein 1	0.022	0.007	3.335	528	Q9NUX5	4.88E-20	Q9NUX5	1.89E-19
protein b4	0.027	0.027	1.001	492	P15308	2.41E-12	P15308	1.47E-13
protein cwc15 homolog	0.012	0.01	1.193	516	Q6IQU4	2.95E-27	Q6IQU4	1.63E-18
protein lin-9 homolog	0.031	0.018	1.706	372	Q5RHQ8	1.12E-79	Q5RHQ8	2.81E-36
protein lsm14 homolog b	0.029	0.014	2.063	501	Q566L7	1.29E-46	Q566L7	3.57E-34
protein serac1	0.05	0.035	1.403	411	Q5SNQ7	4.94E-60	Q5SNQ7	1.60E-44
ras-related protein rab-11a flags: precursor	0.106	0.028	3.786	246	Q5ZJN2	1.06E-65	Q5ZJN2	5.37E-25
selenoprotein t1a flags: precursor	0.033	0.016	2.056	288	Q802F2	1.95E-80	Q802F2	1.06E-35
synaptotagmin-like protein 2 exophilin-4	0.064	0.046	1.374	246	Q99N50	5.69E-34	Q99N50	1.06E-19
tfiia-alpha and beta-like factor	0.034	0.023	1.444	339	Q9UNN4	1.80E-32	Q9UNN4	2.87E-23
tho complex subunit 1	0.033	0.027	1.21	291	Q96FV9	2.55E-68	Q96FV9	1.49E-42
torsin-1b	0.09	0.011	8.023	258	O14657	4.26E-38	O14657	7.91E-07
transcription initiation factor tfiid subunit 12	0.013	0.01	1.259	501	Q3T174	3.39E-61	Q3T174	1.90E-29
translin	0.008	0.008	1.068	486	Q62348	2.08E-78	Q62348	9.20E-58
transmembrane protein 106b	0.019	0.011	1.817	360	Q1LWC2	1.23E-18	Q1LWC2	1.28E-25
transmembrane protein 50a	0.053	0.035	1.518	279	O95807	1.58E-65	O95807	3.27E-37
trna guanosine-2 -o-methyltransferase trm11 homolog	0.036	0.021	1.704	285	Q05B63	2.25E-57	Q7TNK6	7.43E-34
tumor necrosis factor ligand superfamily member 10	0.151	0.127	1.186	309	P50591	2.46E-15	P50591	1.43E-08
tumor necrosis factor receptor superfamily member	0.12	0.105	1.14	309	Q92956	2.24E-28	Q92956	6.81E-13
ubiquilin-4	0.042	0.034	1.234	384	Q99NB8	8.99E-31	Q5R684	3.98E-27
ubiquitin fusion degradation protein 1 homolog	0.011	0.01	1.05	378	Q9ES53	2.56E-98	Q9ES53	3.51E-78
ubiquitin-conjugating enzyme e2 n	0.182	0.136	1.342	171	Q9EQX9	8.30E-36	Q9EQX9	3.51E-07
ubiquitin-like modifier-activating enzyme atg7	0.031	0.013	2.269	276	O95352	4.22E-63	Q5ZKY2	6.44E-33
upf0420 protein c16orf58	0.034	0.029	1.188	435	Q96GQ5	3.11E-40	Q499P8	5.73E-29
vacuolar protein sorting-associated protein 16 homolog	0.016	0.012	1.312	477	Q5E9L7	2.96E-110	Q5E9L7	1.89E-91
wd repeat-containing protein 5	0.121	0.08	1.513	288	Q2KIG2	2.23E-27	Q2KIG2	3.23E-45
zinc finger cchc domain-containing protein 4	0.044	0.02	2.155	450	Q66IH9	2.42E-67	Q6DCD7	1.19E-33
zinc finger hit domain-containing protein 3	0.01	0.009	1.194	408	Q9CQK1	4.83E-24	Q15649	8.10E-24
zona pellucida sperm-binding protein 4 flags: precursor	0.048	0.042	1.136	402	Q12836	1.35E-22	Q12836	1.78E-18

Bold face indicates a significant Fisher’s exact test (*p*-value < 0.05)

### PAML analyses and zona pellucida phylogeny construction

From 11 of the 71 annotated genes found to be under positive selection in our first PAML dataset*,* the LRTs conducted showed that there was no significant difference between models M7 and M8. From the second dataset, which contained our two rockfish species of interest as well as S. *caurinus* and *S. rastrelliger*, only two out of the four were identified to be under adaptive evolution (M8 was significantly different from M7 when using the LRTs). The two genes were FKB12 and TM50a, which contained five and four sites under positive selection respectively. In our third dataset that was composed of five ZP genes from our two rockfish species, *Oreochromis niloticus* and *Oryzias latipes* did not demonstrate signatures of positive selection according to our LRTs analysis (Table 2).

**Table 2:** PAML analyses of candidate and ZP genes with M7 & M8 models

Species	Gene ID	Ka/Ks	EST length	M7 vs. M8	Sites under selection
*S. goodei, S. saxicola, S. caurinus, and S. rastrelliger*	fkb12	1.342	201	14.733	22 (0.997**), 45 (0.952*), 48(0.971*), 53 (0.997**), and 67 (0.992**)
*S. goodei, S. saxicola, S. caurinus, and S. rastrelliger*	r19	1.432	300	Not-Significant	N/A
*S. goodei, S. saxicola, S. caurinus, and S. rastrelliger*	taf12	0.372	231	Not-Significant	N/A
*S. goodei, S. saxicola, S. caurinus, and S. rastrelliger*	tm50a	2.163	279	20.773	90 (0.996*), 91 (0.977*), 92 (0.977*), and 93 (0.958*)
*S. goodei, S. saxicola, Oryzias latipes, and Oreochromis niloticus*	cox5a	0.04	297	Not-Significant	N/A
*S. goodei, S. saxicola, Oryzias latipes, and Oreochromis niloticus*	cp058	0.252	294	Not-Significant	N/A
*S. goodei, S. saxicola, Oryzias latipes, and Oreochromis niloticus*	cwc15	0.067	336	Not-Significant	N/A
*S. goodei, S. saxicola, Oryzias latipes, and Oreochromis niloticus*	if2g	0.116	420	Not-Significant	N/A
*S. goodei, S. saxicola, Oryzias latipes, and Oreochromis niloticus*	ino1a	0.142	456	Not-Significant	N/A
*S. goodei, S. saxicola, Oryzias latipes, and Oreochromis niloticus*	ls14b	3.62	396	Not-Significant	N/A
*S. goodei, S. saxicola, Oryzias latipes, and Oreochromis niloticus*	pri2	0.376	333	Not-Significant	N/A
*S. goodei, S. saxicola, Oryzias latipes, and Oreochromis niloticus*	sirt5	0.174	297	Not-Significant	N/A
*S. goodei, S. saxicola, Oryzias latipes, and Oreochromis niloticus*	tm50a	0.214	231	Not-Significant	50 (0.994**)
*S. goodei, S. saxicola, Oryzias latipes, and Oreochromis niloticus*	tsn	0.129	237	Not-Significant	N/A
*S. goodei, S. saxicola, Oryzias latipes, and Oreochromis niloticus*	znhi3	0.222	282	Not-Significant	N/A
*S. goodei, S. saxicola, Oryzias latipes, and Oreochromis niloticus*	zpax	0.295	477	Not-Significant	N/A
*S. goodei, S. saxicola, Oryzias latipes, and Oreochromis niloticus*	zpb	0.248	663	Not-Significant	N/A
*S. goodei, S. saxicola, Oryzias latipes, and Oreochromis niloticus*	zpc1	0.368	567	Not-Significant	N/A
*S. goodei, S. saxicola, Oryzias latipes, and Oreochromis niloticus*	zpc4	0.437	315	Not-Significant	N/A
*S. goodei, S. saxicola, Oryzias latipes, and Oreochromis niloticus*	zpc5	0.275	483	Not-Significant	N/A

M7 and M8 models were compared with the likelihood ratio test and Ka/Ks values were averaged between the two models. Sites that were found under positive selection are presented with only the Bayes Empirical Bayes (BEB) analyses. Posterior probabilities are labeled as * and ** for *P* > 95 % and *P* > 99 %, respectively

**Fig. 2:**
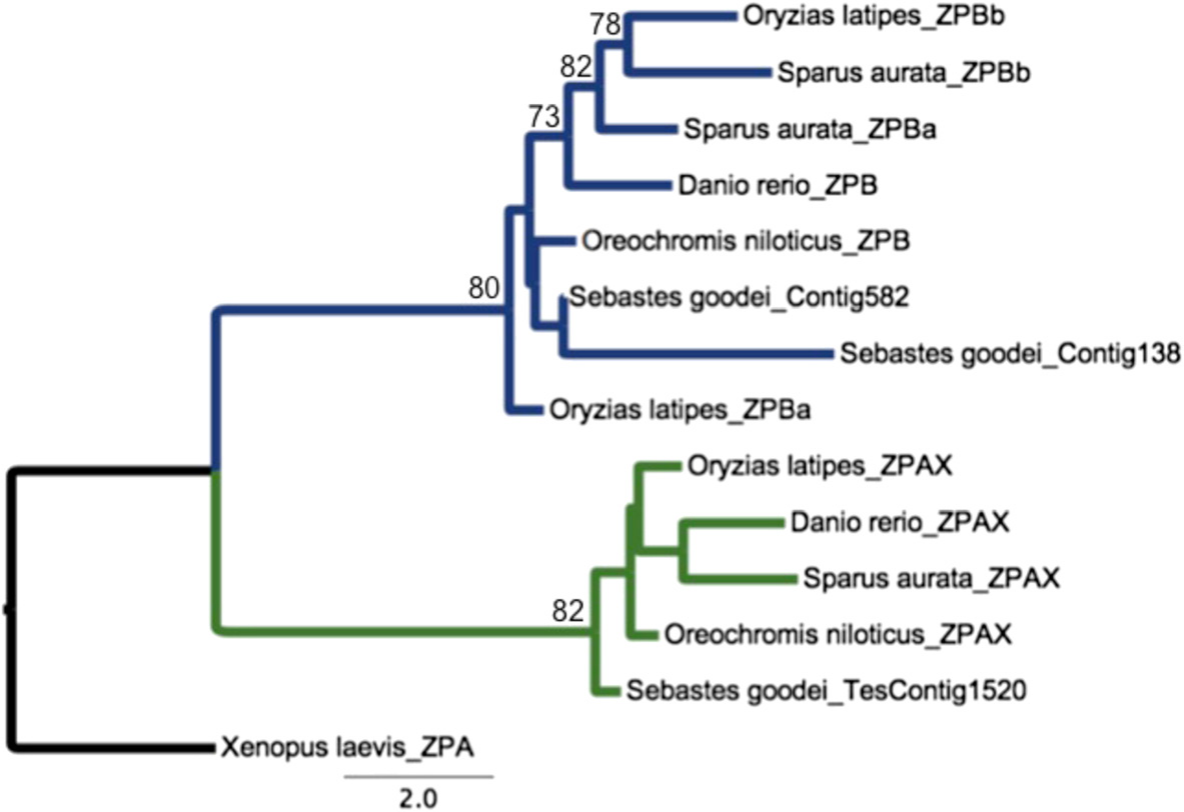
ML tree generated for ZPAX and ZPB genes found within *S. goodei* and *S. saxicola* with 1000 bootstrap replicates. Additional teleost species were used to construct this phylogeny, and bootstrap values greater than 70 are displayed

**Fig. 3:**
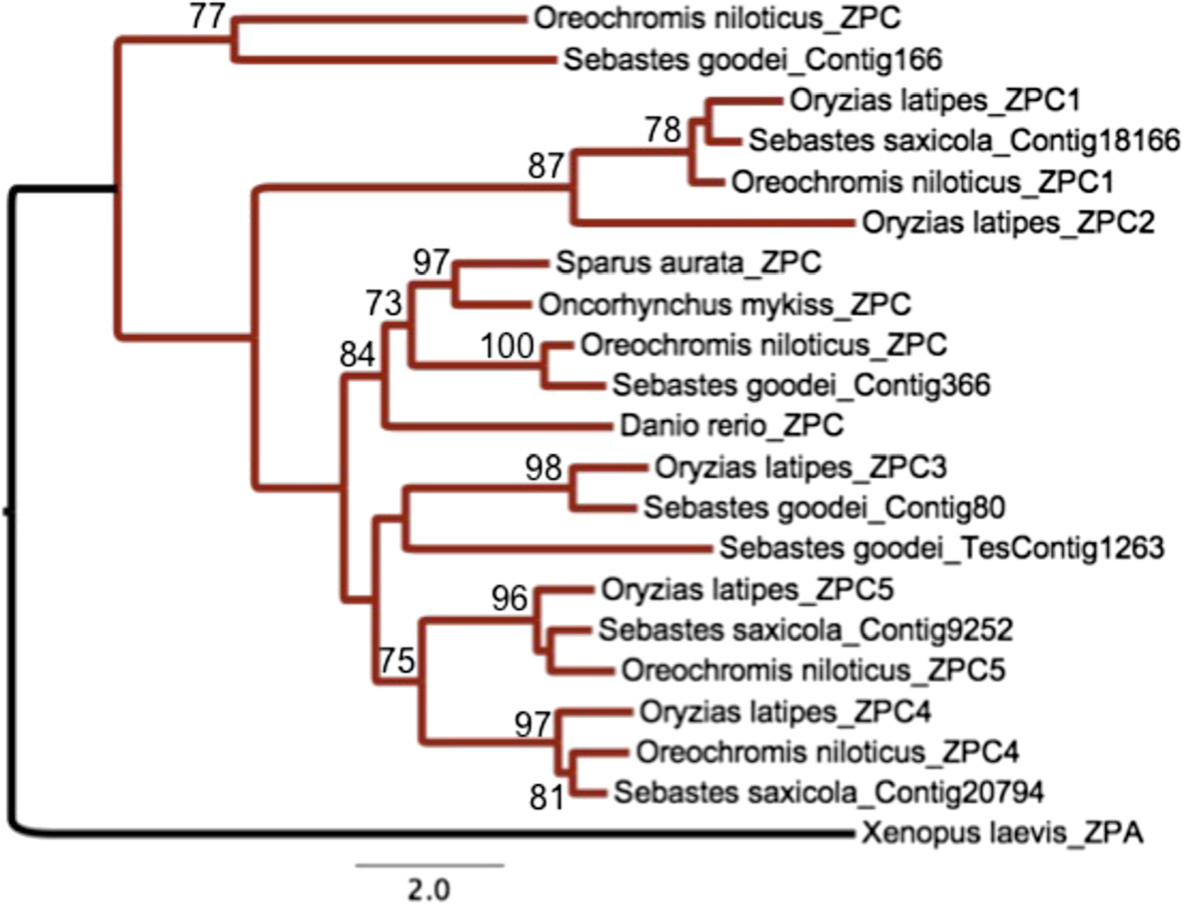
ML tree generated for ZPC genes found within *S. goodei* and *S. saxicola* with 1000 bootstrap replicates. Additional teleost species were used to construct this phylogeny, and bootstrap values greater than 70 are displayed

In our construction of the gene family for ZP within rockfishes we first identified 18 and 26 ESTs that contained ZP annotations for *S. goodei* and *S. saxicola* respectively. Maximum likelihood (ML) trees were constructed with 143 ungapped a.a. sites (1075 total sites) and 92 ungapped a.a. sites (697 total sites) for the ZPAX and ZPB, and ZPC respectively (Figs. 2 and 3). In our phylogenetic analysis, seven ZPC, 2 ZPB, and one ZPAX homologs were identified (Figs. 2 and 3). Some ESTs were excluded from this analysis (two ZPB fragments), because these fragments did not align with the majority of the remaining sequences, however, they were included in the Ka/Ks analysis. From the PAML analysis, five ZP ortholog groups were compared. This was based on the ortholog groups identified (*Sebastes* sequences, *Oryzias latipes*, and *Oreochromis niloticus*). The Ka/Ks comparison was conducted with ten ZP genes (six ZPC pairs, three ZPB pairs and one ZPAX pair), where only one pair (ZPB homolog) was identified under positive selection (Table 3).

**Table 3:** Pairwise Ka/Ks estimates for ZP ortholog pairs

ZP ID	*S. goodei* EST ID	*S. saxicola* EST ID	Method	Ka	Ks	Ka/Ks	Nuc. length
ZPAX	TesSgooContig1520	Contig7124	YN	0.008	0.028	0.304	468
ZPB	SgooContig582	Contig2319	YN	0.007	0.015	0.435	663
ZPB homolog 1	TesSgooContig1769	Contig9672	YN	0.048	0.042	1.136	402
ZPB homolog 2	SgooContig184	Contig10146	YN	0.003	0.03	0.11	402
ZPC homolog 1	SgooContig366	Saxicola_C47406	YN	0.011	0.061	0.186	342
ZPC homolog 2	SgooContig166	Contig6798	YN	0.009	0.034	0.274	957
ZPC1	SgooContig100	Contig18166	YN	0.002	0.013	0.187	558
ZPC3	SgooContig80	Contig9633	YN	0.005	0.021	0.253	525
ZPC4	SgooContig309	Contig20794	YN	0.005	0.012	0.381	300
ZPC5	SgooContig179	Contig9252	YN	0.006	0.026	0.223	471

Bold face indicates an ortholog pair that is found under positive selection

### UTR divergence

Based on 1079 pairwise comparisons (orthologous pairs) between the two rockfish species the average Ka was 0.034 (s.d. = 0.053) and an average Ks value of 0.067 (s.d. = 0.077) by using the YN model. The untranslated region (UTR) divergence estimates between the two fishes were based on 311 and 192 pairwise comparisons for 5’ and 3’ UTRs respectively. The 5’ UTR estimates with a Jukes-Cantor correction were 0.026 (s.d. = 0.025) and Ks values (Jukes-Cantor correction) from the corresponding coding sequences was 0.063, (s.d. = 0.068). The 3’ UTR average was 0.023 (s.d. = 0.024) from the 193 corresponding coding sequences and contained an average Ks value of 0.076 (s.d. = 0.089). Overall, the means for the UTRs were statistically less than the means from the Ks values and there were no clear relationships between UTRs and Ks values. In a pair-wise simple *t*-test and the Wilcoxon rank sum test there were only two comparisons that did not show any mean differences (5’ UTR ends vs. 3’ UTR ends, and 5’ Ks from coding regions vs. 3’ Ks from coding regions - Table 4).

**Table 4:** Pairwise analyses of sequence divergence

*Analysis*	*T test P-value*	*Wilcoxon Rank sum test P-value*
Ks 3prime vs. UTR 3prime	*9.25E-14*	*< 2.2E-16*
Ks 3prime vs. UTR 5prime	*8.48E-13*	*< 2.2E-16*
Ks 3prime vs. Ks 5prime	0.104	0.505
UTR 5prime vs. UTR 3prime	0.207	0.145
Ks 5prime vs. UTR 3prime	*9.27E-20*	*< 2.2E-16*
Ks 5prime vs. UTR 5prime	*2.57E-18*	*< 2.2E-16*

Bold face indicates a significant *P*-value

## Discussion

This study identifies genes under positive selection between the gonadal transcritomes of two distantly related rockfish species (*S. goodei and S. saxicola*). 1079 orthologous gene pairs were identified between the two species and of these we found 144 genes under positive selection. Genes found under positive selection did not overlap with the genes found in a previous *Sebastes* comparative transcriptome study [46], which is not surprising given the differences in the tissue types. However, we did find similar functional traits (metabolism, immune function, and longevity) for genes under selection. In the earlier study, the transcriptomic analysis was conducted with brain, pituitary, kidney, and spleen tissues, which may differ in expression patterns from gonadal tissues. Gene expression among different tissue types is still being teased apart, as genes once thought to be expressed in a tissue specific fashion have been identified in multiple tissues [50]. In addition, the examination of a set of candiate genes from the zona pellucida gene family did not reveal strong signs of positive selction, as has been found in other vertebrates. Lastly, we used divergence estimates of the UTRs to further support that orthologs were identified for our study and not paralogs. As more rockfish tissue-specific transcriptomic information becomes available, the determination of whether certain genes subject to positive selection belong to specific tissues can be determined. This information allows us to better understand how reproductive genes have contributed to the process of adaptive radiation within this group of fishes.

### Comparison of the two datasets

The combination of Sanger and 454 sequencing technologies have been beneficial for increasing the amount of transcriptomic information available for non-model species [51]. In rainbow trout (*Oncorhynchus mykiss*), the combination assembly of Sanger and 454 sequencing showed high similarities with other fish species that have their genomes sequenced [51], which provides support that the combination of the two technologies do not generate disparities or conflicting information. Caveats seen with 454 sequencing is that singletons contained elevated insertions in mycorrhizal fungi [52], and also high errors rates have been found within homopolymer repeats [53]. We did not include singletons in our study and we saw very similar annotations for the two datasets. In addition, we were able to obtain a substantial number of orthologs between the two species datasets (1079 pairs) which suggests that the different sequencing technologies did not hinder our analyses.

### Natural selection

Our scan for genes under positive selection also includes genes with elevated Ka values (Ks = 0) that contained GO terms that were associated with adult life spans and gamete function/production. Genes with only nonsynonymous substitutions and assigned with GO terms associated with gamete production/function were the t-complex protein 1 and lissencephaly-1 homolog. Study on zona pellucida – 3 (homologous to ZPC in fishes) and the t-complex protein 1, and immune system protein *β*_*2*_*m* in a group of closely related murine species (genus *Mus*) contain sites under positive selection [54]. T-complex protein 1 is expressed during spermatogenesis in murids [48], but the specific function is still unknown. This gene is highly expressed within mouse testes and is suggested to maintain normal spermatogenesis. Lissencephaly-1 has been demonstrated to be conserved [55] when compared between mice and humans. This gene has been shown to demonstrate infertility when a homozygous mutant has been developed [56]. The likely scenario for elevated Ka values found in these genes is because these are only fragments of the entire gene sequence. These genes would be interesting to examine at the population level within each respective species in order to determine whether there is variation found at both synonymous and nonsynonymous sites.

Ortholog pairs under positive selection with a Ka/Ks > 1 and GO terms associated with gamete production/function were deadenylating nuclease, DNA ligase III, DNA mismatch repair protein, eukaryotic translation initiation factor 2, and homolog subfamily a member 4 (Table 1). DNA repair mechanisms have a strong relationship with gametogenesis, where the genomes of gametic cells are subject to mutations following recombination [57]. Within these gametic cells the repair mechanisms have to tolerate mutations that occur during gametogenesis which result in specialized functions [57] that are possibly due to selective pressures. Deadenlyating nuclease has been suggested to silence maternal mRNA during oocyte formation [58], this is particularly interesting due to the transcript comparison between *S. goodei* testes and *S. saxicola* ovary tissue. Homolog subfamily a member 4 is known to be part of the DnaJ family, which is assigned to the structurally unrelated protein family of Heat Shock Proteins (HSPs) [59]. In humans, this gene is expressed in brain tissue, but many homologs within the family are associated with sperm motility. Recent study has shown there are differences in reproductive genes between infertile vs. fertile human males, in which DnaJ subfamily A was represented [60]. Clearly, these genes need to be further investigated to understand the mechanistic and functional properties within rockfishes to understand how these genes are subjected to positive selection.

Within our scan for positively selected genes we identified genes associated with longevity. Although the two species have similar lifespans, there are extensive differences between life spans across species within the genus [61], and genes associated with longevity were identified within our previous study [46]. The congener closely related to *S. goodei* is *S. paucispinis* [13], which can live to at least 46 years [61]. By comparison, the nearest congener to *S. saxicola* is *S. semicinctus,* which can live up to 15 years [43]. The genes identified here and associated with longevity were eukaryotic translation initiation factor 2 subunit 3, cytochrome c oxidase subunit 5a, 40s ribosomal protein x isoform, and 60s ribosomal proteins L9 and L17, and protection of telomeres protein 1 [62–64]. These genes associated with longevity are particularly interesting and hold the potential key for understanding how aging operates in this group of fishes. As more rockfish genomic information becomes available this will provide a clearer depiction of the patterns of longevity and how this may impact adaptation.

The genes that showed evidence of positive selection in our PAML analysis were 12-kDa FK506-binding protein (FKBP12) and transmembrane protein 50a (TM50a). FKB12 is known to be associated with various cellular functions that include apoptosis, cell-cycle progression, and calcium release [65]. Genes that encode for the mechanisms of apoptosis have been suggested to be under positive selection [2]. Speculation for why these genes are under selection is due to the genomic conflict that would occur as a result of apoptosis during spermatogenesis [66]. As for TM50a, this gene encodes for a membrane protein and the function of this gene within fishes is unknown. There is more information needed to determine how these genes contribute to adaptation within *Sebastes*.

Other comparative transcriptomic analyses of candidate systems for adaptive radiations, such as crater lake cichlid fishes [1] and East African cichlid fishes [67], showed a limited number of genes found under positive selection, which was less than 1 % and ~ 2.7 % respectively, (both were less than what we found in our study ~ 13.3 %). From these studies on cichlids, some of the genes under positive selection that were comparable to our study were: transmembrane protein, cytochrome c oxidase, lipid phosphate phosphohydrolase, ribosomal proteins from Baldo et al. [67] and RNA-binding from Elmer et al. [1]. These genes would be of interests to investigate further since they are found under positive selection within multiple examples of adaptive radiations, which includes our study.

Currently, there is much debate over the assessment of natural selection at the molecular level. However, one of the limitations to these analyses are that current statistical methods estimate Ka/Ks across an entire gene and does not account for the relaxation of purifying selection, and/or the effects of population bottlenecks [68]. In addition, estimates of Ka/Ks demonstrate a conservative estimate of positive selection, because most of the protein is under a functional constraint and only a few amino acid sites would be subject to positive selection [2]. However, within many comparative genomic studies there are genes that have been identified under positive selection which encode for proteins with immune or reproductive functions [4, 69]. Although there may be difficulties detecting selection, there are reoccurring gene functions that are subject to positive selection. Within our study, we have identified certain genes under positive selection that encompassed a broad range of GO terms where a majority of terms include: cellular process, metabolic process, biological regulation, response to stimulus, multi-cellular organ process, cellular component organization, developmental process, localization, signaling, and reproduction. The specifics about how these genes under positive selection contribute to adaptation within heterogeneous environments remains unknown, but provides a suite of candidates for understanding why these genes have been identified as nonsynonymous substitutions in comparison to the remainder of the transcriptome.

### Zona pellucida

Current evidence shows that there are six subfamilies of zona pellucida genes in vertebrates (ZPA/ZP2, ZPB/ZP4, ZPC/ZP3, ZPD, ZPAX, and ZP1) [38] and these are homologous with the ZP domain found within invertebrates [70]. Our phylogenetic construction of the ZP family suggests there is only one ZPAX gene, two (putatively four) ZPB homologs, and seven (ZPC/ZP3) homologs in our dataset. Most ZP genes within the rockfish genome grouped with *Oreochromis niloticus* and *Oryzias latipes,* which suggests these genes have arisen in a similar pattern from a recent common ancestor (Figs. 2 and 3).

In our estimation of Ka/Ks of ten ZP gene pairs most pairs contained a broad range of Ka/Ks values (Table 3) with only one ortholog pair that was subject to positive selection (ZPB homolog 1). Both ZPB homologs (1 and 2) were not used to construct phylogenetic trees because these sequences provided limited phylogenetic information (weak bootstrap support) and were shorter than the sequences used for our phylogenetic analyses. However, these genes are divergent from the remaining ZP homologs and an ortholog from one of the model teleost could not be detected. It is unknown if some of these ZP homologs are specific to the *Sebastes* lineage, where more information from species within this genus and closely related genera or families would be needed to make this assessment. Currently, there is no evidence of teleost ZP genes subject to positive selection [71], however this was assessed with a select few model fishes (i.e. *Danio rerio*, *Oryzias latipes*, *Gasterosteus aculeatus*, *Tetraodon nigroviridis*, and *Takifugu rubripes*). This poses the question of whether there is enough evidence to show that ZP genes do not provide evidence for positive selection within teleost or is there some other mechanism that would prompt reproductive barriers? These methods are more stringent at identifying selection and the addition of more taxa from *Sebastes* can provide insight on how these genes have contributed to the radiation within this group.

### UTR analysis

**Fig. 4:**
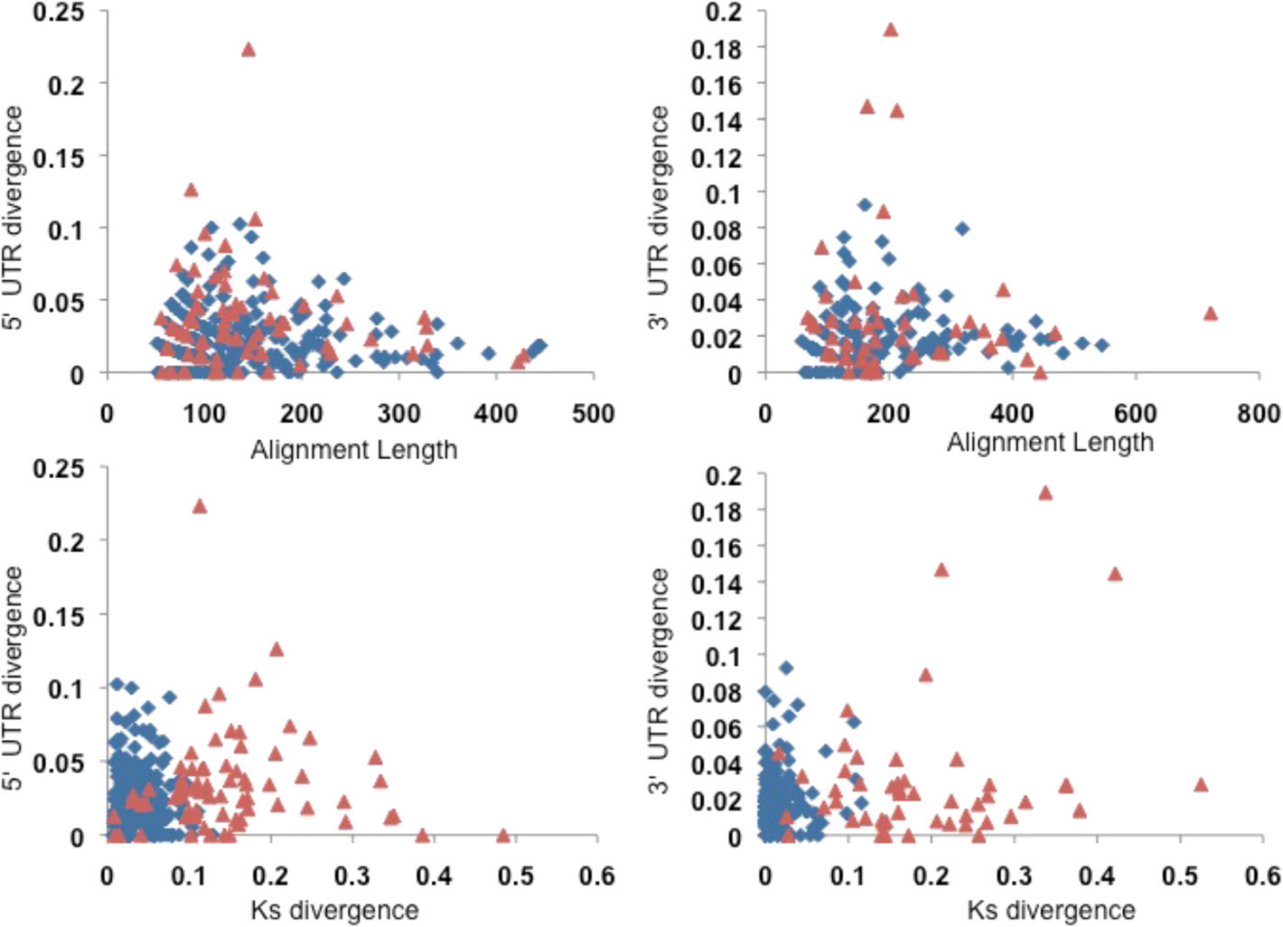
Comparison of UTR divergence with alignment length and Ks divergence. Blue diamonds indicate ortholog pairs with a Ks > 0.1, whereas red triangles indicate Ks values that are greater than 0.1 and less than 0.5

Untranslated regions (UTRs) provide a reference of divergence between species and can be utilized as a base for comparing synonymous substitutions within coding regions that are assumed to be evolving neutrally. Our estimation of 3’ and 5’ UTR divergence is unprecedented within the genus *Sebastes.* Our estimated values of UTR divergence between *S. goodei* and *S. saxicola* were not statically similar to the Ks values (from 5’ and 3’ sequences, Table 4). In addition, the utilization of the cutoff mark (Ks < 0.1) is not an essential benchmark for the removal of aligned pairs as putative paralogs according to our UTR analysis (Fig. 4). Interestingly, the Ks coding region and 3’ UTR divergence between crater lake cichlid fish species contained rates of 0.0250 and 0.0252 (with a Jukes-Cantor correction) respectively which had a common ancestor ~ 10,000 years ago [1, 72]. This provides an interesting comparison of freshwater (cichlids) and marine fishes (rockfishes), where UTR divergence was similar between cichlids and rockfishes but Ks values were different. Hurst [73] suggesteded that synonymous rates are relatively proportional to the neutral mutation rate, which suggests that the UTRs and Ks are relatively close to this rate. However with species that are more divergent, there are distinct differences between synonymous rates and UTR divergence. Divergence within closely related *Drosophila* species are distinct where 3’ UTR and 5’ UTR rates are lower than synonymous sites when comparing *D. melanogaster* and *D. simulans* [74], which diverged ~ 2–3 mya [75]. Our study did not have similar Ks and UTR rates as compared to the Elmer et al. [1] study, which may be due to the amount of time since divergence (estimated 6 mya). Suggestions have been made that lower UTR divergence in comparison to synonymous sites in *Drosophila* is likely to be subject to negative selection, which is consistent with our findings [74]. This pattern of 3’ UTRs subject to purifying selection has also been identified within chimpanzees and humans [76]. More evidence will be required to demonstrate the impact of negative selection on the marine rockfish genome, which analyzing the UTRs from closely to distantly related congeners can provide insight on this evolutionary pattern.

The use of the UTRs has been a useful indicator for assigning the correct ortholog pairs as opposed to paralogs, in addition to the algorithms used in inparanoid [77]. Depending on the function of the gene, UTRs can be highly conserved between orthologs and divergent between paralogs once a gene duplication event has occurred, which has been demonstrated between humans and mice [78]. One of the many difficulties of identifying orthologous gene pairs within teleosts is the proposed fish specific genome duplication (FSGD) event which occurred ~ 350 mya [79]. This event provides a plethora of gene duplicates that may operate under different evolutionary pressures such as subfunctionalization, neofunctionalization, and pseudogenization. With this magnitude of gene duplicates, the assignment of orthologous gene pairs can be difficult because of the amount of duplicates that are closely related. In our study, we showed lower rates of divergence within the UTR region in comparison to the synonymous sites of these two species. If we constructed an alignment of UTRs from a pair of paralogs in which the paralogs arose due to the FSGD, then there would be an expected high degree of divergence as opposed to the divergence rate of true orthologs. However, exceptions may occur with recent gene duplications and/or concerted evolution permits for paralogs to be subjected to similar selective pressures. If we can detect novel genes within this genus we can gain a better perspective of the rate of divergence occurring within the UTR region. Understanding the importance and evolutionary patterns of novel genes is a promising avenue with the advent of next-generation sequencing.

## Conclusions

This transcriptomic study between *S. goodei* and *S. saxicola* provides a template for understanding evolutionary processes at the molecular level within *Sebastes*. We identified a series of candidate genes that are useful for the assessment of the critical genes that diverged and are responsible for the radiation within this group. Genes that pertain to longevity hold potential for understanding the molecular mechanisms that have contributed to the radiation within this genus. The establishment of genes under positive selection from this study can be insightful and utilized to assess whether these positively selected genes are under selection across the entire genus *Sebastes*. If these genes are under positive selection across the entire genus, this will provide new clues about how natural selection is contributing to speciation by reproductive isolation within this group. This study was intended to further advance the field of evolutionary biology by providing support of which functional genes are important for adaptation and sexual selection. With transcriptomic data from multiple species within *Sebastes*, we can identify the repeated patterns of adaptive evolution and elucidate our understanding of how adaptation and the speciation processes occurred across the entire genus of *Sebastes*.

## Methods

### EST sequencing and assemblies: *S. goodei*

A portion of the ovary and testes were collected from fresh dead *S. goodei* individuals (one per sex), placed immediately in RNAlater, and stored at −80 °C. The National Oceanic and Atmospheric Administration (NOAA) Fisheries, Southwest Fisheries Science Center, Santa Cruz, California collected samples under a salvage permit. DNA (cDNA) isolation and library construction was performed by BIO S&T (Montreal, Canada). Total RNA was extracted with TRIzol (Invitrogen, Carlsbad, CA), and cDNA was synthesized according to the SMART cDNA library construction kit (Clontech, USA). The resulting cDNAs were full-length enriched, and possess SfiI A&B at the 5’ and 3’ ends which facilitated directional cloning. Double-stranded cDNAs were obtained by primer extension. Double-stranded cDNAs were digested with Sfi-I, afterwards only fragments greater than 0.5 kb were purified with a gel purification kit.

Purified cDNA was ligated to SfiI-digested and Calf intestinal phosphatase (CIPed) vectors by overnight incubation at 16 °C. The ligation mixture was desalted and electroporated in ElectroMax DH10B cells (Gibco-BRL, USA). Quality control (average cDNA insert sizes and recombinant rate) was performed prior to mass transformation. Transformed cells were distributed into 96-deep-well plates for amplification at about 2300 recombinants per well.

Cells were plated onto LB-agar (amplicillin and x-gal) plates. Clones were prepared for sequencing in two ways. Method 1 – positive colonies were picked directly into 96-well plates that contained LB broth + ampicillin. Cultures were grown overnight at 37 °C with moderate shaking. The Montage Plasmid Miniprep HTS kit (Millipore) was used to isolate plasmid DNA. Sequencing on purified plasmid DNA was done with M13 (−20 and +40) primers at JGI, which was conducted in another study [80]. Method 2 – cDNA libraries were produced by double-stranded cDNA, which was size fractionated to obtain long reads. Afterwards, cDNA inserts were cloned into the vector pExpress1 (Express Genomics, Frederick, MD), and electroporated into *E. coli* strain DH10B. Libraries contained ~ 96 % recombinants with an average insert size of 1.95 kb. Libraries were sequenced on 96-well capillary sequencing platforms (ABI 3700) located at the DOE Joint Genome Institute (JGI, Walnut Creek, CA) and at the Genome Core Facility at the University of California, Merced, CA.

Expressed Sequence Tags (ESTs) were cleaned and assembled with an automated pipeline (EST2uni) [81], which includes base calling (phred), vector trimming and low quality bases removal with lucy [82], and repeat masking with repeatmasker-open 3.0 [83]. Afterwards, the assembly of sequencing reads into unique consensus sequences (unigenes) [81] was conducted with cap3 [49], and functional annotations were conducted with blast [84], in which the hits are then parsed so that a description is listed for each unigene. The unigene datasets are composed of high quality and clean sequences, which are assembled into contigs and singletons [81]. These *S. goodei* sequences can be found at Genbank with the following accession numbers [Genbank: JZ693907-JZ704944]. Unigenes were processed again with cap3 to correct for putative assembly errors and then used for the comparative transcriptomic analysis against the *S. saxicola* dataset.

### EST sequencing and assemblies: *S. saxicola*

Ovary tissue was collected from a single fresh dead *S. saxicola* individual, placed immediately in RNAlater, and stored at −80 °C. The *S. saxicola* individualwas also collected by NOAA Fisheries, Southwest Fisheries Science Center, Santa Cruz, California under a salvage permit. Complementary DNA (cDNA) isolation and library construction for 454 sequencing was performed by BIO S&T (Montreal, Canada). The library was sequenced at the University of South Carolina Environmental Genomics Core facility on a Roche 454 sequencer. The library was sequenced on a ½ of a titer plate.

The *S. saxicola* raw reads and base quality information from the 454 GS FLX sequencing run were first extracted and clipped using the sff_extract 0.2.8 [85] script. Further removal of adaptors and contamination, such as low quality bases and poly (A) stretches, was achieved by using snowhite 1.1.4 [86], a pipeline that implements aggressive cleaning with seqclean (http://sourceforge.net/projects/seqclean/) and tagdust 1.12 [87]. Reads were then processed through repeatmasker-Open 3.0 using the cross_match (Downloaded June 2010; [88]) search engine to search the “teleost fish” database and mask repetitive elements. A primary *de novo* assembly was initially done using the 454 default settings in mira 3.2.0 [89] with a minimum percent identity of 94 %. A secondary assembly was performed on the contigs produced from mira 3.2.0 and all remaining singletons in cap3. A minimum overlap of 25 bp and a minimum %ID of overlap of 95 % was used in the secondary assembly. Finally all contigs less than 300 bp in length were removed before additional analyses.

### Annotation of the *S. goodei* and *S. saxicola* datasets

Both EST datasets were annotated in blast2go [90] with the following Blast parameters: blastx to the swiss-prot database [91], an E-value of 1.0 x E^−6^, 20 blast hits, and a High Scoring Pair length cutoff of 33 nt. The annotation parameters were an *E*-value hit filter of 1.0 x E^−6^, annotation score cutoff of 55, and a gene ontology (GO) weight of 5. A two-tailed Fisher’s Exact Test was used in blast2go to determine whether there was enrichment of GO terms for the orthologous pairs that contained a Ka/Ks > 0.5 in comparison to orthologous pairs that were conservative (Ka/Ks < 0.1).

### Detection of orthologs from the *S. goodei* and *S. saxicola* datasets and estimation of selection

blastx (NCBI blast version, 2.2.17) from the standalone blast package [84] was used to identify homologs in both *S. goodei* and *S. saxicola* ESTs against the swiss-prot database (downloaded June 2011) with five teleost datasets from fugu (*Takifugu rubripes*), medaka (*Oryzias latipes*), green spotted pufferfish (*Tetraodon nigroviridis*), stickleback (*Gasterosteus aculeatus*), and zebrafish (*Danio rerio*) in the Ensembl database [92] (Ensembl 63). Afterwards the blastx reports and the EST sequences were processed through orfpredictor [93], which identifies putative open reading frames and translates nucleotide sequences into protein sequences. The translated protein datasets from *S. goodei* and *S. saxicola* were used in inparanoid 4.0 to identify orthologs and avoid the inclusion of paralogs. *Danio rerio* (Ensembl dataset, Zv9) was used as an outgroup for removing potential false orthologs. Orthologous pairs were aligned based on the putative open reading frame using pal2nal 12.2 [94] and Perl scripts that include clustal w 2.0.10 [95]. Ka and Ks were calculated for the orthologous pairs between *S. goodei* and *S. saxicola* in kaks_calculator 1.2 [96] by using the YN model [97].

### Ortholog identification and positive selection

**Fig. 5:**
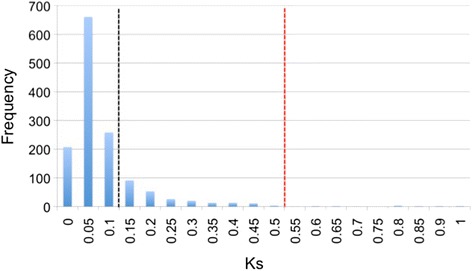
Frequency of ortholog pairs with synonymous substitution estimates. The black dotted line indicates the traditional cut off line and the red dotted line indicates our new threshold cut-off

We used 5336 and 18,505 contigs from *S. goodei* and *S. saxicola* ESTs respectively for the identification of orthologs and the Ka/Ks analyses. There were 1559 orthologs detected with inparanoid 4.0. Once processed through kaks_calculator1.2, pairs were removed from our analyses if the alignment length was less than 150 bp and/or the Ka/Ks values were greater than 50. Ortholog pairs with a Ks value less than 0.1 were further analyzed, which has been used as a benchmark to avoid inclusion of paralogs [98]. We also included a second set of ortholog pairs with Ks values within the range of 0.1–0.5 (Fig. 5). This allowed us to determine whether the Ks > 0.1 benchmark should be extended for our analyses.

### PAML analyses and zona pellucida phylogeny construction

We analyzed three different datasets, in which we tested for adaptive evolution with the paml4.4 [99] software package. We used codeml which is part of the paml4.4 package and tested for positive selection with M7 (neutral model) and M8 (selection model) [100] and conducted Likelihood Ratio Tests (LRTs) between the two models. We conducted a tblastx search with additional datasets from *Oreochromis niloticus*, *Oryzias latipes*, *Sebastes rastrelliger*, and *Sebastes caurinus* to identify orthologs. Only ortholog pairs of length 65 codons or greater, and 85 % identity were utilized for our analysis. The first dataset consisted of orthologs from *Oreochromis niloticus* (Nile Tilapia), *Oryzias latipes* (Medaka) and the two focal *Sebastes* species, which contained eleven genes. Orthologs were identified for genes with elevated Ka/Ks values. These two model species were chosen due to their close relationship to rockfishes when we analyzed our ZP phylogenies. The second dataset included additional orthologs identified from a previous study (*S. caurinus* and *S. rastrelliger*; 46) to further validate signatures of adaptive evolution within the genus that contained four gene pairs.

A third dataset contained sequences from the zona pellucida (ZP) gene family with 5 gene pairs. Sequences annotated to this family were used to construct a phylogeny of the ZP gene family and a fine-scaled analysis of positive selection. *S. goodei* and *S. saxicola* sequences were trimmed and translated within orf predictor. Based on the annotations (assignment of ZP subfamily), the longest ESTs from the two *Sebastes* species were used for phylogenetic analyses and the following subfamilies were identified: ZPAX, ZPB, and ZPC. ZP subfamilies (one sequence alignment dataset for ZPAX and ZPB, and another for ZPC) were aligned with mafft 6 (http://mafft.cbrc.jp/alignment/server/) and a ZPA homolog from *Xenopus levis* was used as an outgroup. These sequences along with teleosts sequences with known ZP annotation [32] and the top tblastx hits from GenBank were translated and aligned in mafft 6 (http://mafft.cbrc.jp/alignment/server/). After alignment, sequences were processed through prottest 3 [101] to determine a model of protein evolution. Phylogenies were constructed with aligned sequences and a selected protein model in PhyML 3.0 [102]. If both a *S. goodei* and *S. saxicola* homologous pair were present, they were processed in kaks_calculator 1.2 by using the YN model [97] to estimate positive selection.

### UTR divergence

We were interested in the neutral substitutional mutation rate within our transcriptomic datasets. In addition, we expected the UTR regions to be highly divergent only if paralogs were identified in our ortholog search between the two datasets. This will give an indication that our Ks cut-off at 0.5 is valid. We developed scripts, which were used to remove 5’ and 3’ UTRs from the orthologous pairs and conduct a pairwise alignment in muscle 3.7 [103]. Lastly, we estimated sequence divergence using a Jukes-Cantor model as suggested by Elmer et al. [1] only pairs greater than 50 bp were used for our analyses. Only pairs that contained both a 5’ and 3’ estimate were removed to prevent a partial paired analysis and we conducted a pair-wise blast of the orthologs to assess the quality of our alignments. blast scores of 90 bits or greater were included for our divergence analysis. Coding regions were reprocessed through kaks_calculator 1.2 and Ks values were estimated with a Jukes-Cantor correction in order to make comparisons. Simple pairwise t-tests and Wilcoxon Rank Sum Tests were calculated between and within coding regions and UTRs by using r (https://www.r-project.org/).

### Availability of supporting data

Raw reads from *S. goodei* were deposited to dbEST under the accession numbers JZ693907-JZ704944. Short reads from *S. saxicola* were deposited to the Short Read Archive under the accession SRR1212396.

## Additional files

Additional file 1:
**EST assembly statististics for** ***S.*** ***goodei.*** (XLS 22 kb) Additional file 2:
***S. saxicola*** **assembly summary statistics.** (XLS 21 kb)
